# Time Trends in Population Prevalence of Eating Disorder Behaviors and Their Relationship to Quality of Life

**DOI:** 10.1371/journal.pone.0048450

**Published:** 2012-11-07

**Authors:** Deborah Mitchison, Phillipa Hay, Shameran Slewa-Younan, Jonathan Mond

**Affiliations:** 1 School of Medicine, University of Western Sydney, Sydney, New South Wales, Australia; 2 School of Medicine, James Cook University, Townsville, Queensland, Australia; 3 Centre for Rural and Remote Mental Health, School of Medicine and Public Health, University of Newcastle, Orange, Australia; 4 School of Sociology, Australian National University, Australian Capital Territory, Canberra, Australia; The University of Hong Kong, Hong Kong

## Abstract

**Objective:**

To examine temporal trends in the burden of eating disorder (ED) features, as estimated by the composite of their prevalence and impact upon quality of life (QoL) over a period of 10 years.

**Methodology:**

Representative samples of 3010 participants in 1998 and 3034 participants in 2008 from the South Australian adult population were assessed for endorsement of ED features (objective binge eating, extreme dieting, and purging were assessed in both years; subjective binge eating and extreme weight/shape concerns were also assessed in 2008) and QoL using the Medical Outcomes Study Short Form (SF-36).

**Principal Findings:**

From 1998 to 2008 significant increases in the prevalence of objective binge eating (2.7% to 4.9%, p<0.01) and extreme dieting (1.5% to 3.3%, p<0.01), but not purging, were observed. Lower scores on the SF-36 were significantly associated with endorsement of any of these behaviors in both 1998 and 2008 (all p<0.001). No significant difference was observed in the effect of the endorsement of these ED behaviors on QoL between 1998 and 2008 (all p>0.05). Multiple linear regressions found that in 1998 only objective binge eating significantly predicted scores on the mental health summary scale of the SF-36; however, in 2008 extreme weight/shape concerns, extreme dieting, and subjective binge eating were also significant predictors. Objective binge eating and extreme dieting were significant predictors of scores on the physical health summary scale of the SF-36 in both 1998 and 2008.

**Conclusions and Significance:**

The prevalence of ED behaviors increased between 1998 and 2008, while their impact on QoL remained stable. This suggests an overall increase in the burden of disordered eating from 1998 to 2008. Given that binge eating and extreme dieting predict impairment in QoL, the necessity of interventions to prevent both under- and over-eating is reinforced.

## Introduction

Although most of the research has focused on young white females from Western cultures, disordered eating is recognized to affect a wide proportion of the world population, and to be present across a wide array of demographic variables. Eating disorder behaviors, such as binge eating, dietary restriction, and purging (self-induced vomiting, laxative misuse, diuretic misuse) for instance have been documented in all age groups [1], and in ethnic minority groups, including African, Hispanic [2], and Indigenous groups [3]. Studies have also found reports of eating disorders in clinical and population based samples in developing countries [4], [5]. Furthermore, disordered eating, in particular binge eating, has been found to be as common in men as in women [6], [7], [8].

Disordered eating is not only evident across a wide range of demographics, it is also quite common [1], [9], [10], [11]. Point prevalence estimates generated from population surveys in the United States [12] and Australia [3] for instance are as high as 4.6% for at least weekly strict dietary or fasting behavior, 1.5% for at least weekly purging, and 7.2% for at least weekly objective binge eating (or 2.1% for the 1 year prevalence of at least twice-weekly objective binge eating). Evidence has also emerged that these behaviors have been increasing in recent years. Comparisons of prevalence rates in sequential population surveys conducted in 1995 and 2005 of men and women in Australia for instance have found that regular objective binge eating and purging increased around two-fold while regular strict dieting and fasting increased around three-fold over a ten year period [1], [13].

In estimating the population burden of disordered eating, impact on current functioning is to be determined as well as prevalence [14], [15]. Most of the research that has measured quality of life, as an indicator of impact, in participants who report disordered eating has found health-related quality of life to be poor in comparison to other people in the population or healthy controls [16]. What remains unclear however is that despite the documented recent increase in the prevalence of disordered eating [1] little is known about whether the impact of disordered eating has increased or decreased. For as long as the change in impact remains unclear, despite sequential prevalence statistics being available, any potential change in the overall burden of disordered eating over time cannot be estimated.

Of particular interest in its impact on population burden is binge eating. Binge eating has been consistently shown to be the most prevalent of the eating disorder behaviors [11], [17], is equally likely to be reported by men as women, and is relatively evenly spread across the age groups [1]. Furthermore, binge eating has been strongly associated with impairment in quality of life [16] and obesity [18], [19]. Binge eating thus represents a particularly problematic behavior that not only burdens the individual through reduced quality of life [16], but also burdens the economy through increased demand on health services to treat and manage associated psychological and physiological sequelae [20], [21].

Weight and shape concerns are considered part of the core pathology of eating disorders [22]. Research has shown that when weight and shape concerns are present alongside eating disorder behaviors, impairment in functioning is greater [6]; and that there may be a proportional relationship between the degree of concern and the level of mental and physical disability [23]. Furthermore weight and shape concerns may be more predictive of mental health functioning than eating disorder behaviors [24]. For instance, the presence of weight and shape concerns in participants who binge eat has been found to predict functional impairment to a greater extent than binge eating itself [25]. Furthermore, in a previous study where weight and shape concerns were entered alongside other eating disorder features in a regression predicting days-out-of-role for women, only weight and shape concerns emerged as a significant predictor [6].

There are few community-based studies of prevalence and disability in the eating disorders field. It is now well known however that the vast majority of people who suffer from eating disorders do not present for treatment [26], [27], [28], and as such may not be well represented by clinical samples. Furthermore, the use of community-based studies is especially pertinent in the investigation of quality of life, since quality of life has itself been found to be a predictor of treatment-seeking in individuals with eating disorders [29]. As such, any investigation into the changing burden of disordered eating will be enriched by the recruitment of a community-based sample.

### Aims

The present study aimed to assess the changing burden of binge eating, purging, and extreme dieting over a 10 year period, as reflected in point prevalence estimates and impairment in health-related quality of life. A secondary aim was to examine which eating disorder features, including those behaviors mentioned above as well as extreme weight and shape concerns, predicted health-related quality of life. These aims were achieved through analysis of behavioral and quality of life data collected in sequential cross-sectional surveys of the South Australian adult population in 1998 and 2008.

## Methods

### Hypotheses

We hypothesized that overall the burden of disordered eating will have increased from 1998 to 2008. This was expected to be evidenced by an increase in the prevalence of binge eating, extreme dieting, and purging from 1998 to 2008; as well as no significant reduction in the health-related quality of life associated with these behaviors from 1998 to 2008. In terms of predicting health-related quality of life, we hypothesized that of all the eating disorder features measured, extreme weight and shape concern would emerge as the most significant predictor.

### Design

The Health Omnibus Survey is conducted annually by Harrison Health Research [30], under the auspices of the South Australian Health Commission, and involves face-to-face interviews of a representative sample of the South Australian population. The data in this study was collected from questions that were embedded into two independent cross-sectional Health Omnibus Surveys, conducted in 1998 and 2008. The overall interviews were respondent-based and asked participants a range of both demographic and health-related questions.

### Ethics Statement

In 1998 and 2008 participants gave verbal rather than written informed consent, due to the practicalities of carrying out such a large-scale survey and also the low risk nature of the survey. Both the 1998 and 2008 surveys were approved by the research ethics committee of the Government of South Australia, Department of Health.

### Sample Selection and Interview Procedure

The sample selection and interview procedures were similar in 1998 and 2008. In both years, metropolitan and rural “collector” districts in South Australia were identified based on a probability proportional to size sampling procedure, according to the Australian Bureau of Statistics 2006 Census data. Within each district, 10 dwellings were chosen to conduct interviews in. The person to be interviewed within each dwelling was the person who was older than 15 years and had their birthday most recently. The samples were non-replacement, and up to six visits were made to conduct an interview with the designated participant in each designated dwelling. Interviews were conducted from March until April 1998 for the 1998 survey and from February until July 2008 for the 2008 survey.

### Assessment of Eating Disorder Features

Three types of eating disorder behaviors were assessed: binge eating (objective and subjective), purging, and extreme dieting. The questions in the surveys that were to elicit information regarding the presence of these behaviors from participants were based on diagnostic questions from The Eating Disorder Examination [31], a structured interview used for eating disorder diagnosis. Objective binge eating was assessed in both surveys by asking participants whether they regularly felt that they ate ‘*an unusually large amount of food*’ and at the same time experienced a feeling of being ‘*out of control*’. Subjective binge eating was only assessed in 2008; it was measured similarly to objective binge eating except that the amount of food consumed was defined as not unusually large. Purging was assessed in both surveys by asking participants whether they regularly used laxatives, diuretics (water tablets), or self-induced vomiting as a means to control their weight or shape. Extreme dieting was assessed in both surveys by asking participants whether they have regularly gone on a ‘*very strict diet’* or ‘*hardly eaten anything at all’* in order to influence their weight or shape. The term ‘regular’ used in each of these questions was defined as the behavior having occurred at least once per week over the three months prior to the interview. An additional question in the 2008 survey asked participants the level of importance they placed on weight and/or shape in determining their self-evaluation. Answers to this question were grouped into two categories, no extreme weight and shape concerns (participants who reported none to moderate importance of weight and shape in determining self-evaluation) and extreme weight and shape concerns (participants who reported extreme to supreme importance of weight and shape in determining self-evaluation).

### Assessment of Quality of Life

The Medical Outcomes Study Short Form (SF-36) was used to assess perceived mental and physical health-related quality of life. In the 1998 survey, version 1 of the SF-36 was used (SF-36v1; [32]); and in the 2008 survey, version 2 of the SF-36 was used (SF-36v2; [33]). Both versions yield four physical health subscales (physical functioning, role limitations due to physical health, bodily pain, general health) and four mental health subscales (vitality, social functioning, role limitations due to emotional health, and mental health), which each contribute to composite physical health and mental health summary scales. Scores out of 100 for each scale are transformed to *T*-values with a normative mean of 50 and standard deviation of 10 to aid comparability. Higher scores indicate greater quality of life. The SF-36 is validated for use in Australia [34] and there are norms for Australian and South Australian populations [35], [36]. In an Australian national household study assessing the psychometrics of the SF-36, Chronbach’s alphas for the subscales were reported to range between 0.82 to 0.93 and good subscale item-discriminant and criterion validity were also reported [37]. Although there are minor variations in the wording of items between SF-36v1 and SF-36v2, it is generally accepted that scores can be compared between the versions [38].

### Data Manipulation

Current body mass index (BMI) was calculated based on reported current weight (kg) and height (m) data (BMI = kg/m^2^). A general ‘eating disorder behavior’ variable was computed, and differentiated participants who reported one or more of the eating disorder behaviors measured in both surveys (objective binge eating, purging, dieting) from those who reported none.

### Statistical Analysis

In order to ensure that the samples were demographically representative, data from the 1998 survey were weighted according to the 1996 Census figures whilst data from the 2008 survey were weighted according to the 2006 Census figures. Statistical analysis was performed using the Statistical Package for Social Sciences (SPSS) version 19.0 software for Windows. Demographic variables, namely gender, age, BMI, relationship status, country of birth, residential location, education status, and income were compared between groups using chi-square tests for categorical variables and analyses of covariance (ANCOVAs) for continuous variables. Chi-square and multivariate logistic regression analyses were used to assess the prevalence of behaviors, and the odds ratio between 1998 and 2008. Multivariate analyses of variance (MANOVAs) were used to assess differences on the SF-36 scale scores as a function of reported eating disorder behaviors and year of survey. Age, BMI, income, education, and gender were included as covariates and differences were considered significant at the 0.05 alpha-level in these analyses. Finally, multiple linear regressions, using the backward elimination method, were used to assess if eating disorder features predicted quality of life. Data on objective and subjective binge eating, extreme dieting, purging, and extreme weight and shape concerns were entered as dichotomous predictors in four separate regressions – two for each survey year for both the mental health (MCS) and physical health (PCS) component scales of the SF-36. Gender, age, and BMI were also entered as covariates. Visual inspection of the standardized residuals showed no serious deviations from assumptions of normality, homoscedasticity, and linearity. A significance level of >0.10 was used as the removal criterion for variables in each of the models.

## Results

### Demographics

Chi square analyses and ANCOVAs were conducted to compare demographic variables between survey years. This information is presented in Table 1. Participants in 2008 were significantly older (*F* (1, 6113) = 9.0, *p*<0.01), had a higher body mass index (*F* (1, 5562) = 40.7, *p*<0.001), were more educated (*χ^2^* (7) = 82.1, *p*<0.001), and had a higher income (*χ^2^* (8) = 551.9, *p*<0.001) than participants in 1998. No other significant differences were detected in the other demographics measured.

**Table 1:** Demographics of Study Participants in the 1998 and 2008 surveys.

	1998 survey	2008 survey	
	*n = *3010	*n = *3034	*p*
**Gender**			*ns*
*Female*	1546 (51.4%)	1555 (51.3%)	
*Male*	1464 (48.6%)	1479 (48.7%)	
**Age in Years (** ***M, SD*** **)**	44.3 (18.8)	45.7 (18.9)	<0.01
**Body mass index** * **(** ***M, SD*** **)**	25.6 (4.9)	26.5 (5.3)	<0.001
**Relationship (** ***N*** **, %)**			*ns*
*Married/Defacto*	1851 (61.5%)	1159 (38.5%)	
*Single*	1902 (62.7%)	1132 (37.3%)	
**Country of Birth (** ***N*** **, %)**			*ns*
*Australia*	2266 (75.3%)	2286 (75.3%)	
*Overseas*	744 (24.7%)	748 (24.7%)	
**Education (** ***N*** **, %)**			<0.001
*Tertiary*	356 (11.8%)	552 (18.2%)	
*Trade/Apprenticeship*	373 (12.4%)	391 (12.9%)	
*Diploma/Certificate*	598 (19.8%)	645 (21.2%)	
*Left school but still studying*	205 (6.8%)	158 (5.2%)	
*Still at high school*	137 (4.6%)	169 (5.6%)	
*Left school after age 15*	783 (26.0%)	715 (23.6%)	
*Left school at/before age 15*	558 (18.5%)	403 (13.3%)	
**Income** * **(** ***N*** **, %)**			<0.001
*≤ A40 000*	1484 (49.3%)	842 (27.8%)	
*A40 001–80 000*	834 (27.7%)	777 (25.6%)	
*> A80 000*	247 (8.2%)	793 (26.1%)	

* Body mass index data available for 2741 (91.0%) of participants in the 1998 survey and 2823 (93.0%) of participants in the 2008 survey who reported both weight and height; income data only available for 2565 (85.2%) of participants in the 1998 survey, and 2412 (79.5%) participants in the 2008 survey who reported their annual household income.

### Prevalence of Eating Disorder Behaviors

Multivariable logistic regressions and chi square analyses demonstrated significant increases in regular objective binge eating (χ^2^ (1) = 21.0, *p*<.001) and extreme dieting (χ^2^ (1) = 20.8, *p*<.001) from 1998 to 2008. These behaviors were more than twice as likely to be reported in 2008. Table 2 displays the frequencies and odds ratios of the three behaviors measured, as well as their aggregate, across the 10 year period.

**Table 2:** Prevalence of eating disorder behaviors in 1998 and 2008.

	1998 survey (*n* = 3010)	2008 survey (*n = *3034)			
	*n* (%)	*n* (%)	*OR* * (95% *CI*)	χ^2^	*p*
**Any Behavior**	130 (4.3%)	253 (8.4%)	2.3 (1.8–2.9)	41.4	0.00
**Objective Binge Eating**	80 (2.7%)	149 (4.9%)	2.2 (1.6–3.0)	21.0	0.00
**Extreme Dieting**	46 (1.5%)	101 (3.3%)	2.3 (1.6–3.4)	20.8	0.00
**Purging**	28 (0.9%)	29 (1.0%)	1.3 (0.7–2.5)	0.0	0.91

* OR = odds ratio; CI = confidence interval.

### Health-Related Quality of Life

To obtain a general picture of the impact of disordered eating on quality of life, analyses were conducted comparing participants who reported any of the three eating disorder behaviors to those who reported no behaviors. A MANOVA revealed no significant interactions between survey year and disordered eating on any of the SF-36 scales (see Table 3), suggesting a stable pattern of impact on quality of life across the survey years. The main effect of disordered eating however was significant and revealed that participants who reported any or more of the three eating disorder behaviors had lower SF-36 scores than those who reported none of these behaviors (all *p*<0.001). There was also a significant main effect of survey year, with most of the SF-36 scores being lower in 2008 than in 1998 (all *p*<0.001; except physical functioning subscale, body pain subscale, and physical health component summary scale).

**Table 3:** Comparison of quality of life (SF-36 scores) of participants who reported eating disorder behaviors (EDB) in 1998 and 2008.

	Reported EDB		No Reported EDB			
	1998 (*n = *112)	2008 (*n = *231)	1998 (*n = *2627)	2008 (*n = *2499)	Survey year*EDB	
SF-36 Subscales	*M (SD)*		*M (SD)*		*F*	*p*
Vitality	46.0 (9.3)	44.7 (10.3)	50.6 (9.4)	49.8 (10.1)	0.08	0.78
Social Functioning	45.1 (12.7)	45.0 (12.7)	50.2 (9.7)	49.7 (12.7)	0.58	0.45
Role Limitations due to Emotional Health	46.9 (12.6)	43.6 (12.7)	50.4 (9.8)	49.5 (10.8)	2.24	0.14
Mental Health	46.0 (12.7)	43.0 (12.7)	51.2 (9.2)	49.7 (9.8)	0.87	0.35
Physical Functioning	49.0 (10.5)	49.3 (9.6)	50.2 (9.2)	50.5 (10.0)	3.35	0.07
Role Limitations due to Physical Health	48.3 (10.7)	46.5 (11.1)	50.0 (10.1)	49.5 (10.6)	0.03	0.87
Bodily Pain	45.5 (10.4)	45.5 (10.1)	50.0 (9.7)	50.0 (10.1)	0.78	0.38
General Health	47.0 (11.2)	44.7 (11.2)	50.8 (9.5)	50.0 (9.8)	0.04	0.85
**SF-36 Summary Scales**						
Mental Health Summary	45.4 (12.7)	42.7 (13.4)	51.3 (9.4)	49.5 (10.2)	0.38	0.54
Physical Health Summary	47.9 (11.3)	48.0 (9.4)	49.3 (9.5)	50.2 (9.9)	0.59	0.44

### Predictors of Quality of Life

The final multiple linear regression models on the MCS scores of the SF-36 found objective binge eating to be a significant predictor in both the 1998 and 2008 surveys, while extreme weight and shape concerns, extreme dieting, and subjective binge eating were also significant predictors in the 2008 survey. The final multiple linear regression models on the PCS scores of the SF-36 in the 1998 and 2008 surveys found objective binge eating and extreme dieting to be significant predictors in both years (see Table 4). These disordered eating variables emerged as significant predictors despite covarying for age, BMI, and gender in each analysis.

**Table 4:** Final multiple linear regression models with mental health (MCS) and physical health component scale (PCS) scores as dependent variables in the 1998 and 2008 survey.

Dependent Variable	Predictor Variables	B	SE (B)	95% CI	*B*	*t*	*p*
**1998**							
MCS	Objective binge eating	−7.27	1.18	−9.58, −4.96	−0.12	−6.18	<0.001
	*Gender*	−1.61	0.37	−2.33, −0.89	−0.08	−4.36	<0.001
	*Age*	0.04	0.01	0.02, 0.06	0.08	4.03	<0.001
*R^2^* = 0.03							
PCS	Objective binge eating	−3.16	1.09	−5.31, −1.04	−0.05	−2.92	0.004
	Extreme Dieting	−2.92	1.38	−5.62, −0.23	−0.04	−2.13	0.034
	*Age*	−0.18	0.01	−0.20, −0.16	−0.35	−19.09	<0.001
	*Body mass index*	−0.22	0.04	−0.29, −0.16	−0.12	−6.37	<0.001
	*Gender*	−0.63	0.34	−1.30, −0.05	−0.03	−1.83	0.067
*R^2^* = 0.15							
**2008**							
MCS	Weight/Shape Concerns	−3.30	0.53	−4.34, −2.27	−0.12	−6.26	<0.001
	Objective binge eating	−4.83	1.04	−6.88, −2.79	−0.10	−4.64	<0.001
	Extreme Dieting	−4.42	1.10	−6.57, −2.26	−0.08	−4.02	<0.001
	Subjective binge eating	−4.62	1.65	−7.85, −1.39	−0.06	−2.80	0.005
	*Age*	0.04	0.01	0.02, 0.06	0.07	3.50	<0.001
	*Body mass index*	−0.07	0.04	−0.15, 0.00	−0.04	−1.91	0.057
	*Gender*	0.76	0.41	−0.03, 1.56	0.04	1.89	0.059
*R^2^* = 0.06							
PCS	Objective binge eating	−2.57	0.83	−4.20, −0.94	−0.06	−3.10	0.002
	Extreme Dieting	−1.94	0.93	−3.75, −0.12	−0.04	−2.09	0.037
	*Age*	−0.20	0.01	−0.22, −0.18	−0.37	−20.57	<0.001
	*Body mass index*	−0.28	0.03	−0.35, −0.22	−0.15	−8.44	<0.001
	*Gender*	1.15	0.35	0.48, 1.83	0.06	3.34	0.001
*R^2^* = 0.19							

*R^2^* = variance in the dependent variable accounted for by the predictor variables.

## Discussion

It was hypothesized that the overall burden of disordered eating would have increased from 1998 to 2008. The findings from this study indicated that while there was a non-significant increase in purging in 2008, there was a significant and over two-fold increase in both extreme dieting and objective binge eating. Previous research has also found around a two-fold increase in disordered eating over a 10-year period from 1995 to 2005 [1]. Although there is only three years between ours and this previous study, taken together the findings suggest that there is a steady rate in the increase of disordered eating in the South Australian community.

As stated previously, in order to estimate burden, impact on quality of life as well as prevalence needs to be considered. Our study found that while health-related quality of life associated with disordered eating was not lower in 2008 compared to 1998, the relationship remained stable - that is that quality of life was significantly poorer in those who reported disordered eating. As far as we are aware, our study has been the first to conduct sequential assessments of the impact (as measured by quality of life) of disordered eating in an adult population sample. Overall, our finding of increased prevalence of disordered eating paired with the finding of stable impairment in quality of life suggests that overall the burden of disordered eating is increasing over time.

Extreme weight and shape concerns were hypothesized to emerge as the most significant predictor of health-related quality of life. This turned out to be accurate in 2008, the year in which weight and shape concerns were measured, for mental health but not for physical health-related quality of life. Overvaluation of weight and shape is considered a core feature of the eating disorders. However, while it is present in the diagnostic definitions of anorexia nervosa and bulimia nervosa [22], the criteria for binge eating disorder do not account for weight and shape concerns. This is despite research suggesting a strong association between weight and shape concerns, binge eating, and functional impairment [25], [39], [40].

Of the eating disorder features measured in this study, objective binge eating was found to be the most significant predictor of physical health-related quality of life in 1998 and 2008. Furthermore, objective binge eating was a highly significant predictor of mental health-related quality of life, second only to weight and shape concerns in 2008, and emerging as the sole eating disorder predictor in 1998. These findings support previous research that has shown binge eating to be a debilitating behavior, associated with both psychological and physiological consequences [11], [18], [19].

An interesting finding in this study was that subjective binge eating also emerged as a significant predictor of mental health-related quality of life in the year it was measured, 2008. Subjective and objective binge eating share the core component of a sense of loss of control during the eating binge, while the actual amount eaten differs – being excessive in objective but not subjective binges. These findings give strength to previous work that has found that the element of loss of control, rather than the actual amount eaten, is the strongest predictor of mental health and functioning in participants with binge eating disorder [11], [41] and bulimia nervosa [16], [42]. Recent studies of have also found no differences in health related quality of life associated with objective versus subjective binge eating [43], [44].

On the other hand, this study also found that extreme dieting, a product of too much control over eating, predicted both mental and physical health-related quality of life. This suggests that similarly to loss of control over eating, being overly controlling with dietary intake to the point of extreme dietary restriction is also problematic for wellbeing and disruptive of daily functioning. This finding is in line with previous research that has found dietary restriction to be associated with increased days out of role in adults [6] and depressed mood in adolescents [45].

The internal validity of the current study was strengthened through the rigorous survey procedures used by Harrison Health in the Health Omnibus Surveys, as well as the use of the SF-36, which is a standardized assessment of quality of life. The external validity of the current study was supported by the use of a representative sample of the adult population, and as such we may more confidently be able to extrapolate the present findings to the adult community at large. Of course, external validity was however limited in that the sample came only from one state in Australia, and it would be of interest to see if similar research conducted in future find results similar to those presented in this article.

### Limitations and Future Research

A limitation of the current study is that the time period assessed occurred five years previous to this publication. More recent statistics of prevalence and impact of eating disorder behaviors would be useful to determine whether the trend observed between 1998 and 2008 is continuing or has stabilized. Other limitations pertain to assessment. Firstly, only a select number of eating disorder features were assessed. In particular, there was no assessment of excessive or obligatory exercise behavior. It would be of interest to consider change in prevalence and quality of life impairment associated with such behavior, as well as other cognitive eating disorder features such as preoccupation with weight or shape and fear of weight gain. Secondly, there has been criticism that generic measures such as the SF-36 are insensitive to impairment in the eating disorders [46]. Furthermore, given its generic nature, it is difficult to ascertain whether variance in SF-36 scores is due to disordered eating or to other co-occurring problems. While a disease-specific measure of quality of life in eating disorders would go some way to solving this, these measures also assume that participants are able to accurately partition the source of impairment [6]. A direct comparison of generic and eating disorder-specific instruments would be of interest in future research. Finally, it is also possible that the lack of significant findings involving purging was due to the small number of participants who endorsed this behavior. Future research with larger numbers would clarify whether in fact purging is increasing in the community and what its true impact on quality of life is. However, given the low base rate of purging behaviors, very large sample sizes would be needed to address this issue.

## References

[1] Hay PJ, Mond JM, Buttner P, Darby A (2008) Eating Disorder Behaviors Are Increasing: Findings from Two Sequential Community Surveys in South Australia. PLoS ONE. pp. e1541. 10.1371/journal.pone.0001541 PMC2212110 18253489

[2] Cachelin F, Barzegarnazari E, Striegel-Moore R (2000) Disordered eating, acculturation, and treatment-seeking in a community sample of Hispanic, Asian, Black, and White women. Psychology of Women Quarterly 24: 244–253.

[3] Hay P, Carriage C (2012) Eating disorder features in indigenous Aboriginal and Torres Strait Islander Australian Peoples. BMC Public Health 12: doi:10.1186/1471–2458–1112–1233. 10.1186/1471-2458-12-233 PMC3342121 22439684

[4] Mashhadi A, Noordenbos G (2012) Dieting and the development of eating disorders among Iranian women: Comparing the risk between Tehran and the Netherlands. Food, Culture and Society: An International Journal of Multidisciplinary Research 15: 43–60.

[5] Miller M, Pumariega A (2001) Culture and eating disorders: A historical and cross-cultural review. Psychiatry 64: 93–110. 11495364 10.1521/psyc.64.2.93.18621

[6] Mond JM, Hay PJ (2007) Functional impairment associated with bulimic behaviors in a community sample of men and women. International Journal of Eating Disorders 40: 391–398. 17497705 10.1002/eat.20380

[7] Striegel-Moore R, Rosselli F, Perrin N, DeBar L, Wilson GT, et al (2009) Gender difference in the prevalence of eating disorder symptoms. International Journal of Eating Disorders 42: 471–474. 19107833 10.1002/eat.20625 PMC2696560

[8] Lewinsohn P, Seeley J, Moerk K, Striegel-Moore R (2002) Gender differences in eating disorder symptoms in young adults. International Journal of Eating Disorders 32: 426–440. 12386907 10.1002/eat.10103

[9] Hay P (2003) Quality of life and bulimic eating disorder behaviors: Findings from a community-based sample. International Journal of Eating Disorders 33: 434–442. 12658673 10.1002/eat.10162

[10] Hay P (1998) The epidemiology of eating disorder behaviors: An Australian community-based survey. International Journal of Eating Disorders 23: 371–382. 9561427 10.1002/(sici)1098-108x(199805)23:4<371::aid-eat4>3.0.co;2-f

[11] Mond J, Hay P, Rodgers B, Owen C, Crosby R, et al (2006) Use of extreme weight control behaviors with and without binge eating in a community sample: Implications for the classification of bulimic-type eating disorders. International Journal of Eating Disorders 39: 294–302. 16528679 10.1002/eat.20265

[12] Hudson JI, Hiripi E, Pope HG Jr, Kessler RC (2007) The Prevalence and Correlates of Eating Disorders in the National Comorbidity Survey Replication. Biological Psychiatry 61: 348–358. 16815322 10.1016/j.biopsych.2006.03.040 PMC1892232

[13] Darby A, Hay P, Mond J, Quirk F, Buttner P, et al (2009) The rising prevalence of comorbid obesity and eating disorder behaviors from 1995 to 2005. International Journal of Eating Disorders 42: 104–108. 18949767 10.1002/eat.20601

[14] Mond J, Rodgers B, Hay P, Korten A, Owen C, et al (2004) Disability associated with community cases of commonly occurring eatinq disorders. Australian and New Zealand Journal of Public Health 28: 246–251. 15707171 10.1111/j.1467-842x.2004.tb00703.x

[15] Andrews G, Henderson S, Hall W (2001) Prevalence, comorbidity, disability and service utilisation: Overview of the Australian National Mental Health Survey. British Journal of Psychiatry 178: 145–153. 11157427 10.1192/bjp.178.2.145

[16] Jenkins PE, Hoste RR, Meyer C, Blissett JM (2011) Eating disorders and quality of life: A review of the literature. Clinical Psychology Review 31: 113–121. 20817335 10.1016/j.cpr.2010.08.003

[17] Darby A, Hay P, Mond J, Quirk F, Buttner P, et al (2009) The rising prevalence of comorbid obesity and eating disorder behaviors from 1995 to 2005. International Journal of Eating Disorders 42: 104–108. 18949767 10.1002/eat.20601

[18] Telch C, Agras W (1994) Obesity, binge eating and psychopathology: Are they related? International Journal of Eating Disorders 15: 53–61. 8124327 10.1002/1098-108x(199401)15:1<53::aid-eat2260150107>3.0.co;2-0

[19] Darby A, Hay P, Mond J, Rodgers B, Owen C (2007) Disordered eating behaviours and cognitions in young women with obesity: Relationship with psychological status. International Journal of Obesity 31: 876–882. 17130850 10.1038/sj.ijo.0803501

[20] Simon J, Schmidt U, Pilling S (2005) The health service use and cost of eating disorders. Psychological Medicine 35: 1543–1551. 16219112 10.1017/S0033291705004708

[21] Müller-Riemenschneider F, Reinhold T, Berghöfer A, Willich S (2008) Health-economic burden of obesity in Europe. European Journal of Epidemiology 23: 499–509. 18509729 10.1007/s10654-008-9239-1

[22] American Psychiatric Association (2000) Diagnostic and Statistical Manual for Mental Disorders Washington D.C.: American Psychiatric Association.

[23] Muennig P, Jia H, Lee R, Lubetkin E (2008) I Think Therefore I Am: Perceived Ideal Weight as a Determinant of Health. American Journal of Public Health 98: 501–506. 18235062 10.2105/AJPH.2007.114769 PMC2253567

[24] Mond J, Hay P, Rodgers B, Owen C (2011) Mental health impairment associated with eating-disorder features in a community sample of women. Journal of Mental Health 20: 456–466. 21942682 10.3109/09638237.2011.577112

[25] Mond JM, Hay PJ, Rodgers B, Owen C (2007) Recurrent binge eating with and without the 'undue influence of weight or shape on self-evaluation': Implications for the diagnosis of binge eating disorder. Behaviour Research and Therapy 45: 929–938. 17010307 10.1016/j.brat.2006.08.011

[26] Cachelin FM, Striegel-Moore RH (2006) Help seeking and barriers to treatment in a community sample of Mexican American and European American women with eating disorders. International Journal of Eating Disorders 39: 154–161. 16252278 10.1002/eat.20213

[27] Hart L, Granillo M, Jorm A, Paxton S (2011) Unmet need for treatment in the eating disorders: A systematic review of eating disorder specific treatment seeking among community cases. Clinical Pschology Review 31: 727–735. 10.1016/j.cpr.2011.03.004 21501580

[28] Mond JM, Hay PJ, Darby A, Paxton SJ, Quirk F, et al (2009) Women with bulimic eating disorders: When do they receive treatment for an eating problem? Journal of Consulting and Clinical Psychology 77: 835–844. 19803564 10.1037/a0015336

[29] Mond JM, Hay PJ, Rodgers B, Owen C (2007) Health service utilization for eating disorders: Findings from a community-based study. International Journal of Eating Disorders 40: 399–408. 17497708 10.1002/eat.20382

[30] Health GoSADo (2008) Health Omnibus Survey 2008.

[31] Fairburn CG, Cooper Z (1993) The Eating Disorder Examination (12^th^ Ed.). In: Fairburn CG, Wilson G, editors. Binge Eating: Nature, Assessment and Treatment. New York: Guildford Press.

[32] Ware J, Kosinski M, Keller S (1994) SF-36 Physical and Mental Health Summary Scales: A User's Manual. Boston: The Health Institute.

[33] Ware J, Kosinski M, Dewey J (2000) How to score version 2 of the SF-36 health survey. Lincoln: Quality Metric Inc.

[34] McCallum (1995) The SF-36 in an Australian sample: Validating a new, generic health status measure Australian Journal of Public Health. 19: 160–166. 10.1111/j.1753-6405.1995.tb00367.x 7786942

[35] ABoS (1995) National Health Survey: SF-36 Population Norms, Australia. Canberra.

[36] Hawthorne G, Osborne RH, Taylor A, Sansoni J (2007) The SF36 Version 2: Critical analyses of population weights, scoring algorithms and population norms. Quality of Life Research: An International Journal of Quality of Life Aspects of Treatment, Care & Rehabilitation 16: 661–673. 10.1007/s11136-006-9154-4 17268926

[37] Butterworth P, Crosier T (2004) The validity of the SF-36 in an Australian National Household Survey: demonstrating the applicability of the Household Income and Labour Dynamics in Australia (HILDA) Survey to examination of health inequalities. BMC Public Health 4: 44. 15469617 10.1186/1471-2458-4-44 PMC524495

[38] Studies PRaO (2008) Brief report: The short form 36 version 2: 2008 update. South Australian Department of Health 2008–01.

[39] Hrabosky J, Masheb R, White M, Grilo C (2007) Overvaluation of shape and weight in binge eating disorder. Journal of Consulting and Clinical Psychology 75: 175–180. 17295577 10.1037/0022-006X.75.1.175

[40] Grilo C, Hrabosky J, White M, Kelly A, Stunkard A, et al (2008) Overvaluation of shape and weight in binge eating disorder and overweight Journal of Abnormal Psychology. 117: 414–419. 10.1037/0021-843X.117.2.414 PMC3652231 18489217

[41] Colles S, Dixon J, O'Brien P (2008) Loss of control is central to psychological disturbance associated with binge eating disorder. Behavior and Psychology 16: 608–614. 10.1038/oby.2007.99 18239550

[42] Pratt E, Niego S, Agras S (1998) Does the size of a binge matter? International Journal of Eating Disorders 24: 307–312. 9741041 10.1002/(sici)1098-108x(199811)24:3<307::aid-eat8>3.0.co;2-q

[43] Jenkins P, Conley C, Hoste R, Meyer C, Blissett J (2012) Perception of control during episodes of eating: Relationships with quality of life and eating psychopathologyInternational Journal of Eating Disorders 45. 10.1002/eat.20913 21344466

[44] Mond JM, Latner JD, Hay PH, Owen C, Rodgers B (2010) Objective and subjective bulimic episodes in the classification of bulimic-type eating disorders: Another nail in the coffin of a problematic distinction. Behaviour Research and Therapy 48: 661–669. 20434132 10.1016/j.brat.2010.03.020

[45] Crow S, Eisenberg ME, Story M, Neumark-Sztainer D (2006) Psychosocial and behavioral correlates of dieting among overweight and non-overweight adolescents. Journal of Adolescent Health 38: 569–574. 16635769 10.1016/j.jadohealth.2005.05.019

[46] Doll HA, Petersen SE, Stewart-Brown SL (2005) Eating disorders and emotional and physical well-being: Associations between student self-reports of eating disorders and quality of life as measured by the SF-36. Quality of Life Research: An International Journal of Quality of Life Aspects of Treatment, Care & Rehabilitation 14: 705–717. 10.1007/s11136-004-0792-0 16022064

